# The application of exosomes in the early diagnosis and treatment of osteoarthritis

**DOI:** 10.3389/fphar.2023.1154135

**Published:** 2023-04-28

**Authors:** Anjing Chen, Yangmengfan Chen, Xiao Rong, Xuanhe You, Diwei Wu, Xinran Zhou, Weinan Zeng, Zongke Zhou

**Affiliations:** ^1^ Department of Orthopedics, Orthopedic Research Institute, West China Hospital, Sichuan University, Chengdu, China; ^2^ Department of Scientific Research and Experiment Management, West China Hospital, Sichuan University, Chengdu, China; ^3^ Department of Ultrasound, West China Hospital, Sichuan University, Chengdu, China; ^4^ West China Biobanks and National Clinical Research Center for Geriatrics, West China Hospital, Sichuan University, Chengdu, China; ^5^ West China School of Nursing, Sichuan University/Department of Orthopedics, West China Hospital, Sichuan University, Chengdu, China

**Keywords:** exosome, mesenchymal stem cell, osteoarthritis, inflammation, immune activation, bone

## Abstract

With the increase in human lifespan and the aggravation of global aging, the incidence of osteoarthritis (OA) is increasing annually. To better manage and control the progression of OA, prompt diagnosis and treatment for early-stage OA are important. However, a sensitive diagnostic modality and therapy for early OA have not been well developed. The exosome is a class of extracellular vesicles containing bioactive substances, that can be delivered directly from original cells to neighboring cells to modulate cellular activities through intercellular communication. In recent years, exosomes have been considered important in the early diagnosis and treatment of OA. Synovial fluid exosome and its encapsulated substances, e.g., microRNA, lncRNA, and proteins, can not only distinguish OA stages but also prevent the progression of OA by directly targeting cartilage or indirectly modulating the immune microenvironment in the joints. In this mini-review, we include recent studies on the diagnostic and therapeutic modalities of exosomes and hope to provide a new direction for the early diagnosis and treatment of OA disease in the future.

## 1 Introduction

Osteoarthritis (OA) is a prevalent degenerative joint disease that affects articular cartilage, subchondral bone, and even the entire joints, e.g., knee and hip. This complex disease is characterized by cartilage degeneration, subchondral ossification, and synovitis (Walsh et al., 2023). According to a recent longitudinal study, there were 5.7% of men and 10.3% of women over the age of 60 had symptomatic knee OA (Tang et al., 2016), and there are approximately 15 million newly diagnosed OA patients in the world (Jacob and Kostev, 2021). The pain, stiffness, and disability caused by cartilage damage in joints severely affect the quality of the patient’s life. Although total joint replacement is recognized as the most effective treatment for reducing joint pain and improving joint mobility for OA patients but it is applicable only for end-stage OA patients and has serious complications, e.g., periprosthetic joint infection, pulmonary embolism, deep venous thrombosis (Neuprez et al., 2020). The key to managing and controlling the progression of OA is the proper timing, i.e., early diagnosis and treatment. Unfortunately, few diagnostic modalities and effective treatments exist for the pathological alternations of the articular cartilage on early-stage OA. At present, the treatment of OA patients aims to temporarily alleviate the pain and inflammation by using glucosamine, non-steroidal anti-inflammatory drugs (NSAIDs), physical therapy, or intraarticular injection (Schnitzer et al., 2019), which are symptomatic treatments but do not prevent or reverse the underlying pathological alternations. Therefore, a sensitive diagnostic modality for early-stage OA and an effective treatment to counteract the progression of early-stage OA are major concerns for patients and health insurance systems. In recent decades, a variety of biological therapy has been proposed to treat early-stage OA, e.g., stem cell therapy (McGonagle et al., 2017; Murphy et al., 2020), platelet-rich plasma (Raeissadat et al., 2021), functional biomaterials (Chuang et al., 2023), and tissue engineering technology (Chuang et al., 2023; Duan et al., 2023). Although the above treatments exhibited great therapeutic potential, they also have inevitable limitations respectively. Notably, exosome-based therapy is an emerging treatment and has recently been recognized as a promising way for the early diagnosis and treatment of various diseases, including but not limited to OA.

In this review, we will introduce the advantages of using exosomes for early-stage OA diagnosis and the current applications of exosomes for OA treatment, and point out the critical directions for studies and clinical applications in the future.

## 2 Synovial fluid exosomes serve as the biomarkers for the diagnosis of early-stage OA

The lack of early diagnostic modalities delay the management and prevention of early-stage OA, thus indirectly exacerbating the progression of OA. Clinically, OA is diagnosed by imaging and physical examination, which are relatively inaccurate assessments for early-stage OA as the mild symptoms and unobvious radiographic evidence, e.g., joint space narrowing and the loss of cartilage thickness (Crossley et al., 2008). Considering early-stage OA is basically limited to the joint, the cytokines alternation either lacks specificity or is barely altered in blood (Zhao and Xu, 2018). To figure out a sensitive target for early diagnosis, synovial fluid has attracted attention. Synovial fluid is a type of viscous body fluid that is secreted by the inner layer of synovial membranes and stored in the joint cavity, the earliest pathophysiological hints of OA should appear in the synovial fluid, i.e., secreted exosomes (Skriner et al., 2006). Therefore, synovial fluid exosome is one of the best diagnostic targets for early-stage OA. Molecules and proteins which are distinctively expressed in different stages of diseases thus are considered the essential markers for diagnosis. Considering that few detectable alternations exist in the early-stage OA, the phenotype and components of enriched synovial fluid exosome provide a promising marker for early diagnosis. The function of wrapped substances in the exosome, e.g., messenger RNA, microRNAs (miRNAs), DNA, and proteins, are generally affected by the type and status of the original cell, and subsequently involved in the intercellular communications. Therefore, the above merits endow synovial fluid-derived exosomes with the diagnostic and therapeutic potential for early-stage OA.

In recent years, the diagnostic value of exosomes was intensively explored. For instance, the blood-derived exosomes with a high level of CD47^+^ were recognized as a screening indicator for the breast cancer (Lian et al., 2019; Chen et al., 2022). Similarly, the urinary exosomes can function as a diagnostic index for specific renal diseases (Lee et al., 2023a). Therefore, harvesting the exosomes in the synovial fluid in a minimally invasive way, then analyzing the markers of exosomes may provide a novel and sensitive diagnostic modality for early-stage OA. The Homeobox (Hox) gene, involves the encoding of a transcription factor that aims to modulate limb morphogenesis and bone formation, while the dysregulation of Hox is associated with the initiation and further progression of early-stage OA (Pelttari et al., 2015). As a result, the gene expression of Hox in chondrocyte-derived exosomes in the synovial fluid could be a potential diagnostic modality. In addition, exosome-expressed miRNA is also a specific marker for early-stage OA. It was reported that differentiated hUC-MSCs (human umbilical cord mesenchymal stromal cells) have a high expression of miRNA-140-5 P, which is a downstream effector of SOX9 and involves in the regeneration of the extracellular matrix of cartilage (Geng et al., 2020). The defective interaction between miRNA-140 and Let-7 has been found to cause significant growth defects of chondrocytes in OA disease (Li et al., 2018). Besides, miRNA-145 was reported to be highly expressed in OA patients, it targets Notch signaling and to involve in the apoptosis of chondrocytes (Wang et al., 2020). Interestingly, database analysis identified synovial fluid-derived exosomal miRNA expressed in OA patients in a gender-dependent manner (Kolhe et al., 2020), the differently expressed exosome-derived markers verified the fact that more female OA patients were found than male OA patients. Circular RNA (circRNA) is one of the non-coding RNAs which has a stable closed-loop structure in exosomes (Hsiao et al., 2017). Similar to miRNA, the expression of certain circRNA can also indicate the progression of OA (Wu and Zou, 2021), e.g., OA patients have higher expression of circRUNX2 (Wang et al., 2021) and circCDH13, which can modulate both miRNA-127-5p and MMP13 to aggravate the OA (Li et al., 2019). CircRNA_0032131 was reported to correlate with OA progression by regulating miR-502-5p and PRDX3 (Xu and Ma, 2021). Likewise, circRNA_0005105 involves in the degradation of extracellular matrix in cartilages (Wu et al., 2017), and circ_0008365 is highly expressed in serum-derived exosomes from OA patients and thus showed a diagnostic value for OA patients (Shuai et al., 2022). In addition, exosomal lncRNA PCGEM1 was also highly expressed in OA patients, and it was different in different stages of OA, which suggests that exosomal lncRNA PCGEM1 from the synovial fluid not only served as a marker to diagnose OA but also distinguish the stages of OA (Zhao and Xu, 2018). Furthermore, synovial-derived exosomes can also indicate the inflammatory status of the OA joint in its early stage. Theoretically, various inflammatory cytokines are expressed in synovial fluid, but they are difficult to be directly detected due to the low concentration at the early stage of inflammation (Wang and He, 2018). However, synovial fluid exosomes provide an approach to indirectly indicate the inflammation and identify the OA progression. For instance, IL1R^+^ synoviocyte can be activated after the binding of IL1R and IL-1 from the synovial fluid, and it subsequently secrete specific exosomes (MMP-13^high^ and ADAM5^high^) (Mehta et al., 2019), therefore, the content of exosomes (MMP-13^high^ and ADAM5^high^) may indirectly indicate the IL-1 levels in the synovial cavity. In summary, the phenotypes and wrapped molecules of synovial fluid exosomes can function as the accurate and effective biomarkers for the diagnosis of early-stage OA.

## 3 The exosome-based treatment has multidimensionally therapeutic effects on OA

Exosome also has multidimensionally therapeutic effects on the progression of OA, as they envelop various bioactive substances, e.g., cytokines, growth factors, and RNA, which can be directly transferred in neighboring cells through membrane fusion and then modulate the signal transduction to promote the cell proliferation, differentiation, and matrix formation (Lee et al., 2023b). Thus, the exosome-based treatment can function as an effective therapy to modulate the pathological damage of cartilage for OA patients (Xian Bo et al., 2022).

The therapeutic effects of exosome on OA is basically related to its original cells, e.g., bone mesenchymal stem cells (BMSCs), adipose tissue mesenchymal stem cells (AMSCs), synovial mesenchymal stem cells (SMSCs), and embryonic stem cells (ESCs), because these cells exhibit different cellular activities and biological responses to the different stages of OA (Asghar et al., 2020; Fan et al., 2022). Specific exosomes in the synovial fluid are involved in the expression of collagen type II alpha 1 (Col2A1), and aggrecan (ACAN) in articular cartilage thus alleviating the development of OA (Kato et al., 2014). Modification of bone targeting exosomes with siShn3 to silence Shn3 in osteoblasts enhances new bone formation and inhibits osteoclasts formation by downregulated RANKL and TRAP (Cui et al., 2022). MSC-derived exosome has similar therapeutic effects with its original cells on the treatment of OA, i.e., increasing the expression of chondrogenesis (Col2A1 and ACAN) and inhibiting catabolic enzyme (MMP-13 and ADAMTS5) (Cosenza et al., 2017). Granulocytic-myeloid-derived suppressor cells (GMDSCs)-derived exosomal miRNAs miRNA-29A-3 P and miRNA-93-5 P can effectively reduce arthritis index, leukocyte infiltration, and cartilage destruction in an OA mouse model by inhibiting inflammatory responses of T helper (Th1) cells, i.e., Th1 cells, and Th17 cells (Zhu et al., 2019). Exosomes with a high expression of miRNA-26a-5p can alleviate the injury of synovial fibroblasts induced by prostaglandin-endoperoxide synthase (Rizzi et al., 2022). Exosomal miRNA-100-5 P showed inhibitory effects on the mammalian target of rapamycin (mTOR) and inflammation thus having therapeutic effects on OA (Luo et al., 2019). Likewise, exosomal miR-92a-3p can inhibit WNT family member 5 A (WNT5a) to relieve the cartilage damage caused by OA (Yan et al., 2021). In conclusion, the above studies demonstrated that various exosome-encapsulated miRNAs exhibited multidimensionally therapeutic effects on OA, the application of exosomes can not only directly promote the proliferation, differentiation of chondrocytes, increase the extracellular matrix formation and the regeneration of cartilage, but also indirectly prevent the progression of OA through interacting with surrounding immune cells. Nevertheless, the exploration and selection of an appropriate target gene or molecule still require more systematic and rigorous research in the future.

## 4 Perspectives

**FIGURE 1:**
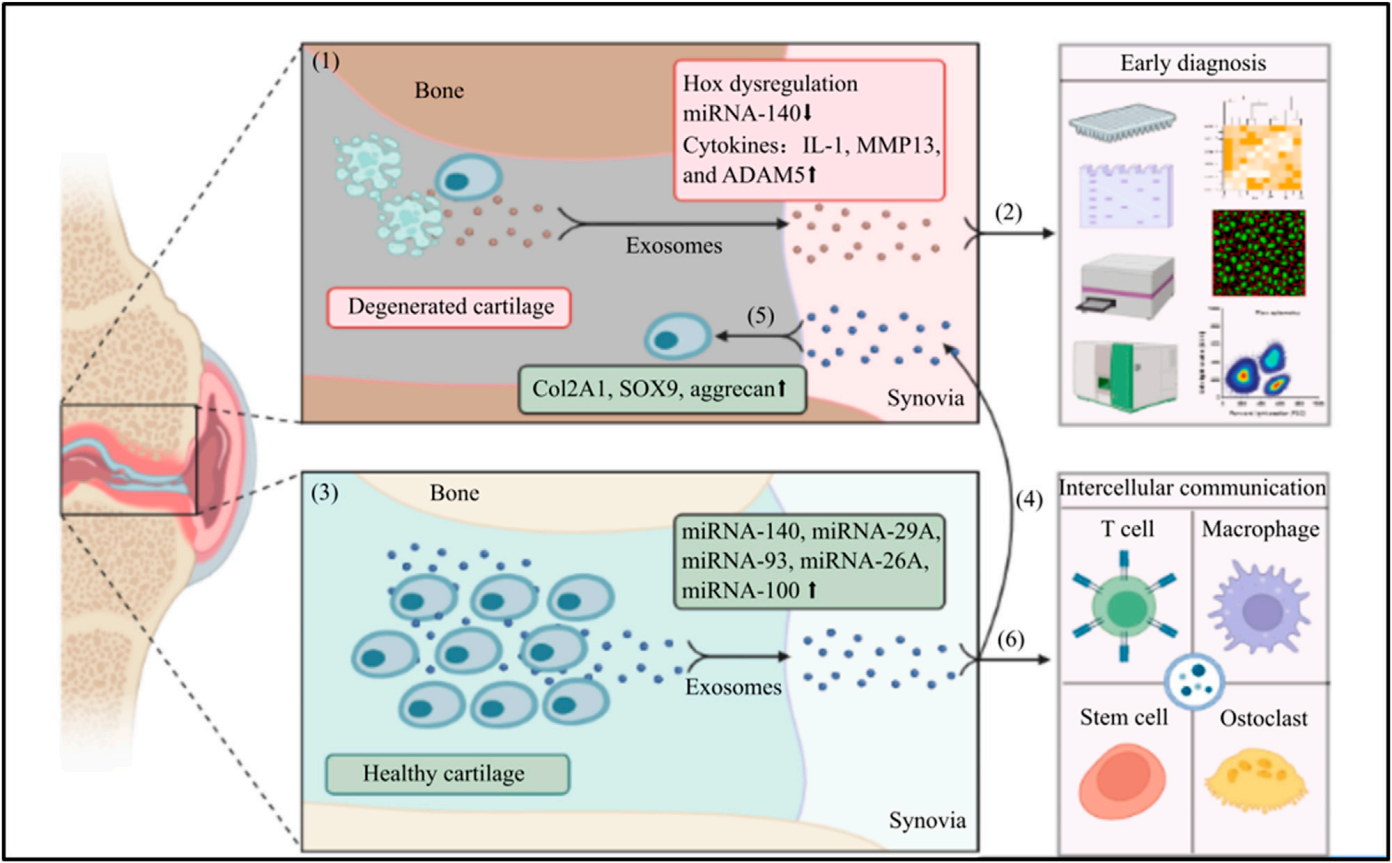
The application of exosomes in the early diagnosis and treatment for OA: (1) In an OA joint, chondrocytes undergo apoptosis, the structure of cartilage is damaged. Chondrocytes and mesenchymal stem cells in the OA environment intend to secrete exosomes with Hox gene dysregulation, low expression of miRNA-140, and high levels of IL-1, MMP13 and ADAM5; (2) The exosomes are secreted into synovial fluid that can be harvested in a minimally invasive way, the specific phenotype or enveloped component of exosomes are sensitive markers to early diagnose OA and differentiate the pathological stages; (3) In contrast, chondrocytes and mesenchymal stem cells in a healthy joint can secrete exosomes with high expression of various miRNA; (4) The exosomes with specific miRNAs are secreted into the synovial fluid; (5) The synovial fluid derived-exosomes can be harvested and injected in to a OA joint, the wrapped functional miRNAs can increase the levels of Col2A1, SOX9, and aggrecan, to promote the regeneration of cartilage and reverse the progression of OA; (6) Meanwhile, the synovial fluid derived-exosomes can also involve in a intercellular communication with many neighboring cells, e.g., T cell, macrophage, osteoclast, and stem cell to multidimensionally protect the joint harmed by OA. (The graphic components of this illustrative scheme were provided by BioRender.com).

The characteristics of exosomes provide us with a novel, sensitive, and effective diagnostic modality and, treatment for early-stage OA (illustrative scheme) (Figure 1). Nevertheless, there still exist many challenges and a long way to the wide clinical utilization, e.g., 1) the short circulating half-life of exosomes limits the systemic application; 2) the targeting ability of exosome-based therapy could be further improved to increase its efficiency and safety. For instance, superparamagnetic iron oxide nanoparticles (SPION) have been well developed to assist in the targeting of exosomes (Zhuo et al., 2021); 3) the efficiency of production, purification, and storage are also key factors that affect its applications; 4) the pathology of OA is complicated that can be affected by various factors, e.g., environment, genic factors, metabolism, or mechanical damage, thus personalized exosomes-based diagnostic modality and therapy should be encouraged. Overall, the advantages of applying exosomes in the early diagnostics and treatment of OA far outweigh its weakness. With the above weakness overcome in the future, exosome-based technology shall be the next-generation of diagnosis modality and treatment for OA.
